# Supplementary material for: A Scoping Review on the Use of Non-Invasive Brain Stimulation Techniques for Persistent Post-Concussive Symptoms
Source: Biomedicines. 2024 Feb 17;12(2):450. doi: 10.3390/biomedicines12020450 (PMC10887310; doi:10.3390/biomedicines12020450)
Supplement: Supplementary file 1 [file biomedicines-12-00450-s001.zip › biomedicines-2826761-supplementary.pdf]

	1. Was the study described as randomized, a randomized trial, a randomized clinical trial, or an RCT?	2. Was the method of randomization adequate (i.e., use of randomly generated assignment)?	3. Was the treatment allocation concealed (so that assignments could not be predicted)?	4. Were study participants and providers blinded to treatment group assignment?	5. Were the people assessing the outcomes blinded to the participants' group assignments?	6. Were the groups similar at baseline on important characteristics that could affect outcomes (e.g., demographics, risk factors, co-morbid conditions)?	7. Was the overall drop-out rate from the study at endpoint 20% or lower of the number allocated to treatment?	8. Was the differential drop-out rate (between treatment groups) at endpoint 15 percentage points or lower?	9. Was there high adherence to the intervention protocols for each treatment group?	10. Were other interventions avoided or similar in the groups (e.g., similar background treatments)?	11. Were outcomes assessed using valid and reliable measures, implemented consistently across all study participants?	12. Did the authors report that the sample size was sufficiently large to be able to detect a difference in the main outcome between groups with at least 80% power?	13. Were outcomes reported or subgroups analyzed prespecified (i.e., identified before analyses were conducted)?	14. Were all randomized participants analyzed in the group to which they were originally assigned, i.e., did they use an intention-to-treat analysis?
De Launay et al.	Quasi-randomized	No	No	Yes	Yes	Yes	Under (0%)	No dropout	Yes (100%)	NR	No	No	No	Yes
Leung et al. (2016)	RCT	Yes	No	No	No	Yes	Under (14%)	NR	Yes (85%)	Similar	Yes	No	No	No
Leung et al. (2018)	RCT	Yes	NR	No	No	Yes	More (34%)	NR	No	Similar	Yes	No	No	NR
Moussavi et al.	RCT	Yes	Yes	Yes	Yes	Yes	Under (18%)	Lower	Yes	Similar	Yes	No	Yes	Yes
Stilling et al.	RCT	Yes	Yes	Yes	Yes	No	Under (0%)	No dropout	Yes	Similar	Yes	No	Yes	Yes

Table S1. Quality Assessment of the Studies based on NIH Study Quality Assessment Tools. The table summarizes the quality assessment process for the included studies based on NIH criteria for quality assessment of controlled intervention studies. NR= not reported.
